# Supplementary material for: MoMCP1, a Cytochrome P450 Gene, Is Required for Alleviating Manganese Toxin Revealed by Transcriptomics Analysis in Magnaporthe oryzae
Source: Int J Mol Sci. 2019 Mar 29;20(7):1590. doi: 10.3390/ijms20071590 (PMC6480321; doi:10.3390/ijms20071590)
Supplement: Supplementary file 1 [file ijms-20-01590-s001.zip › ijms-461402 suppl/Supplementary Figure.docx]

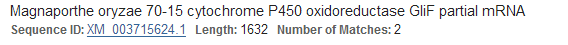

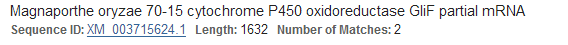

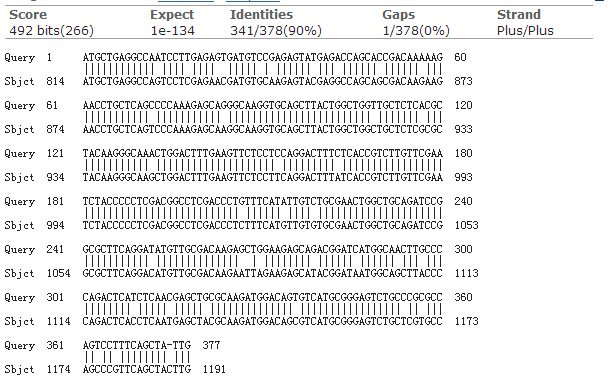

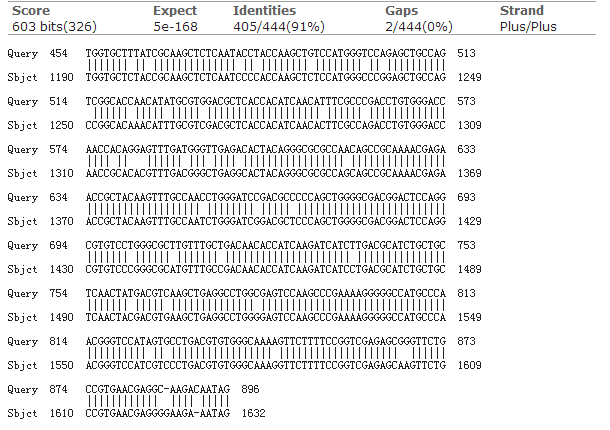


*MoMCP1*

*MoMCP1*

**(A)**


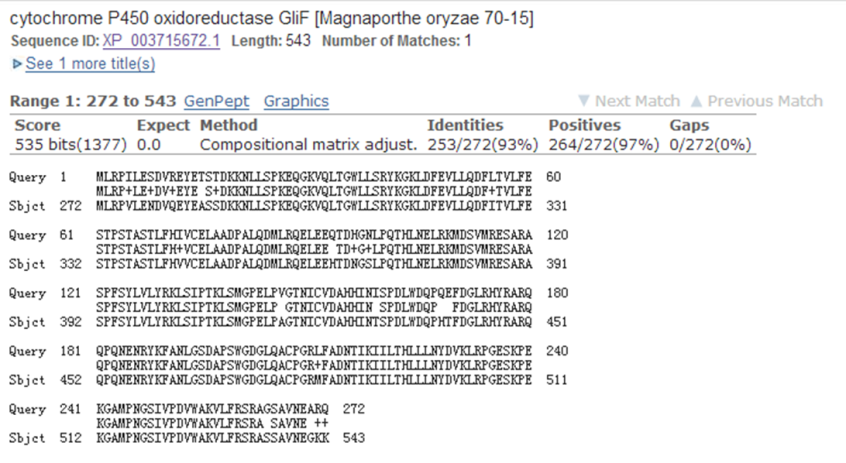


MoMCP1

**(B)**

**Figure S1.** The blast result of MoMCP1 in NCBI. (A) The blast results of DNA sequence in NCBI. (B) The blast results of protein sequence in NCBI.


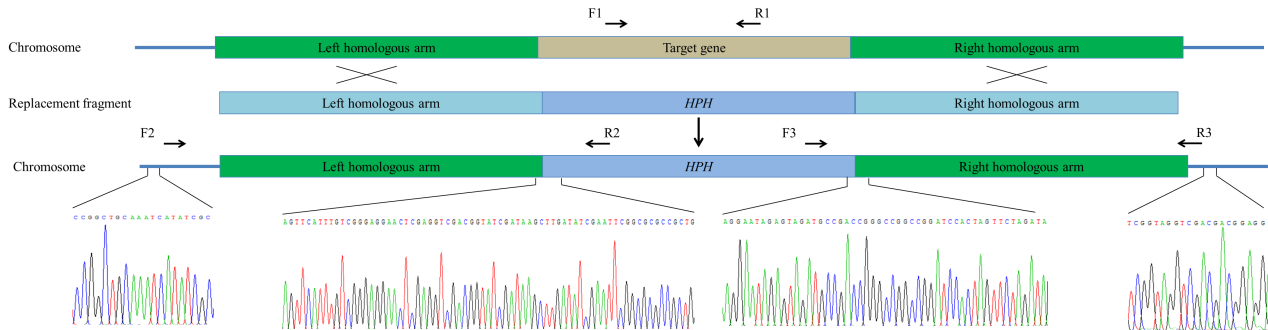


**Figure S2.** The identification of △*Momcp1* by PCR and Sanger sequencing. The sequences of primers are listed in Table S13.

**
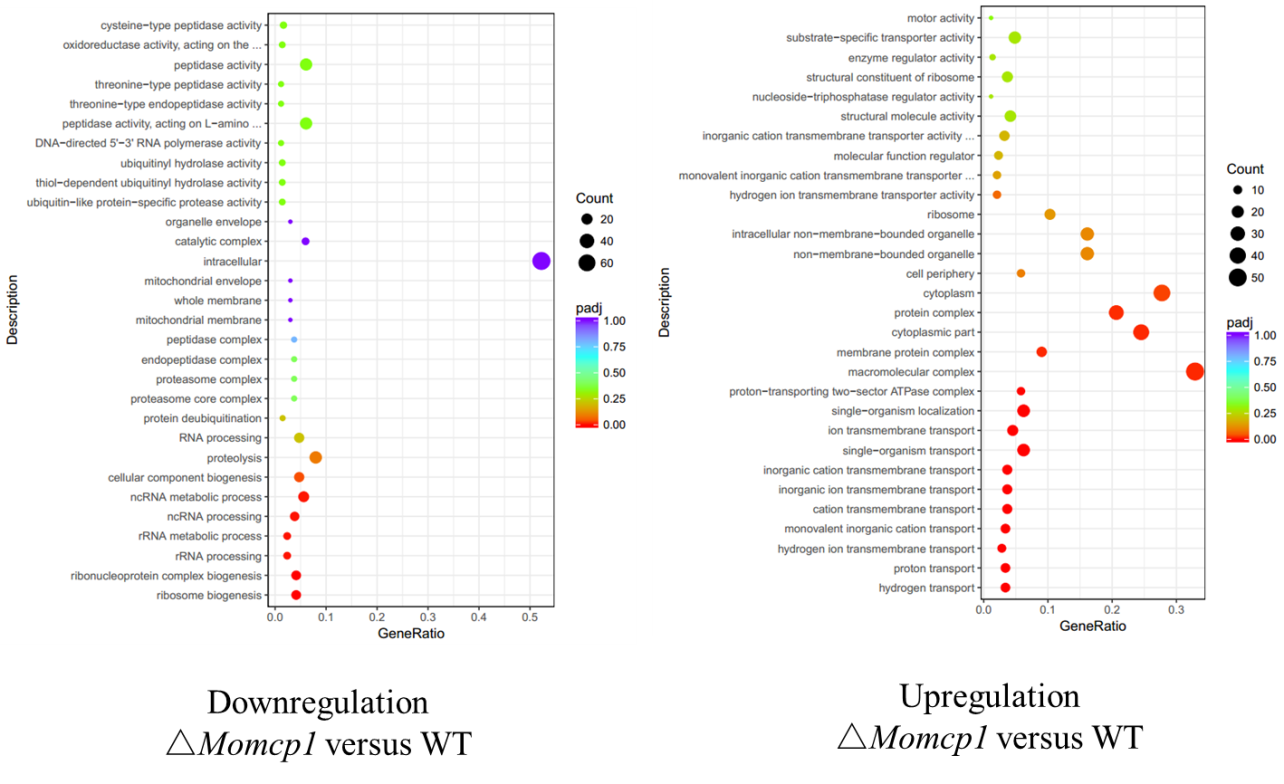
**

(**B**)

(**A**)

**Figure S3.** GO enrichment of DEGs in △*Momcp1* compared with WT. (**A**) GO enrichment about down expressions of genes in △*Momcp1* compared with WT. (**B**) GO enrichment about up expressions of genes in △*Momcp1* compared with WT.


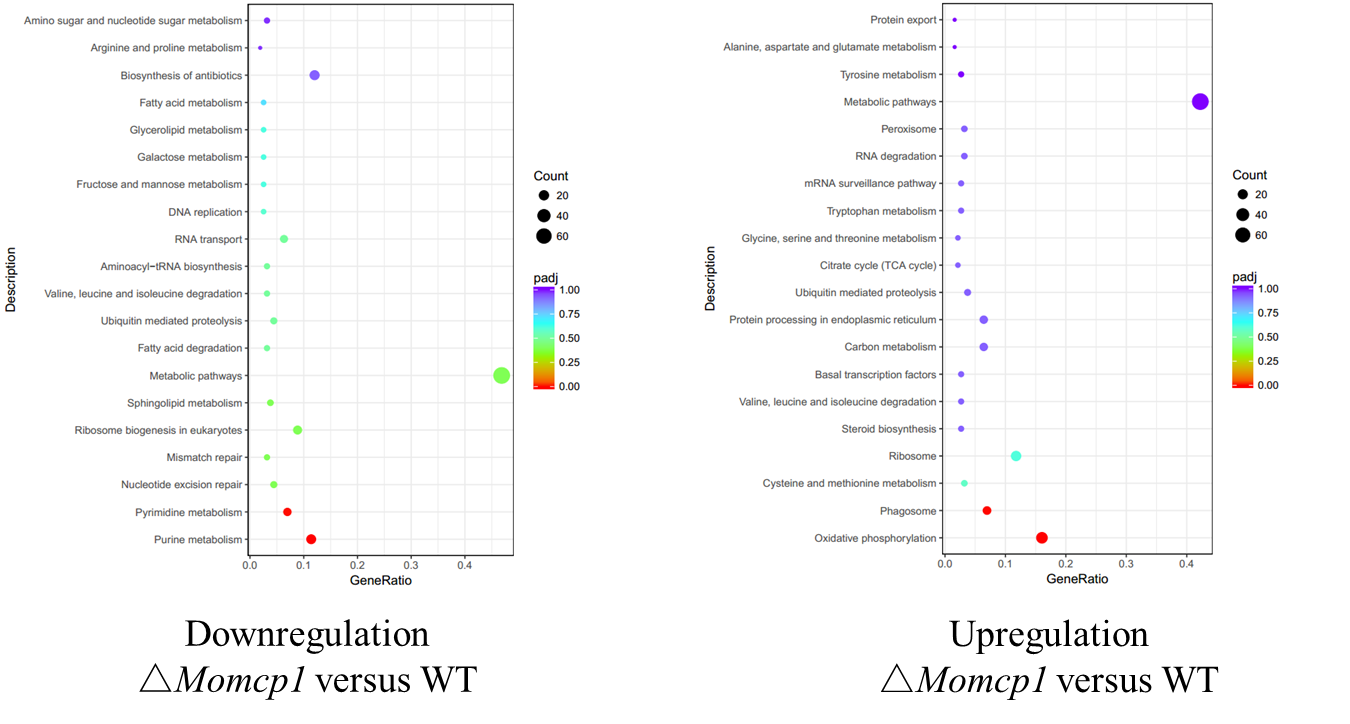


(**A**)

(**B**)

**Figure S4.** KEGG pathways of DEGs in △*Momcp1* compared with WT. (**A**) KEGG pathways about down expressions of genes in △*Momcp1* compared with WT. (**B**) KEGG pathways about up expressions of genes in △*Momcp1* compared with WT.


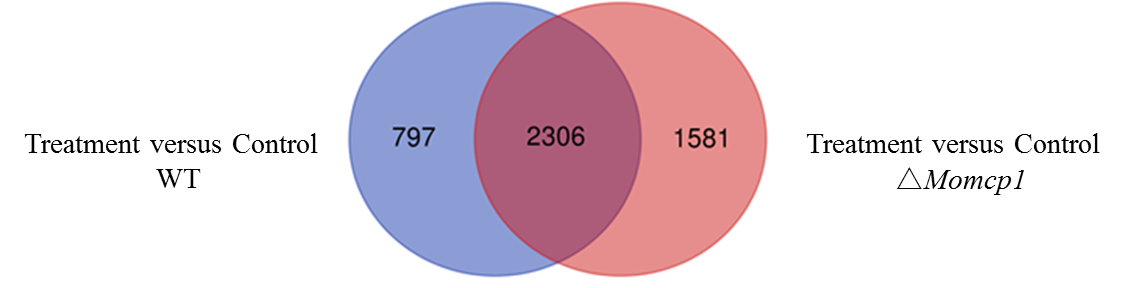


**Figure S5.** The numbers of DEGs in WT and △*Momcp1*.


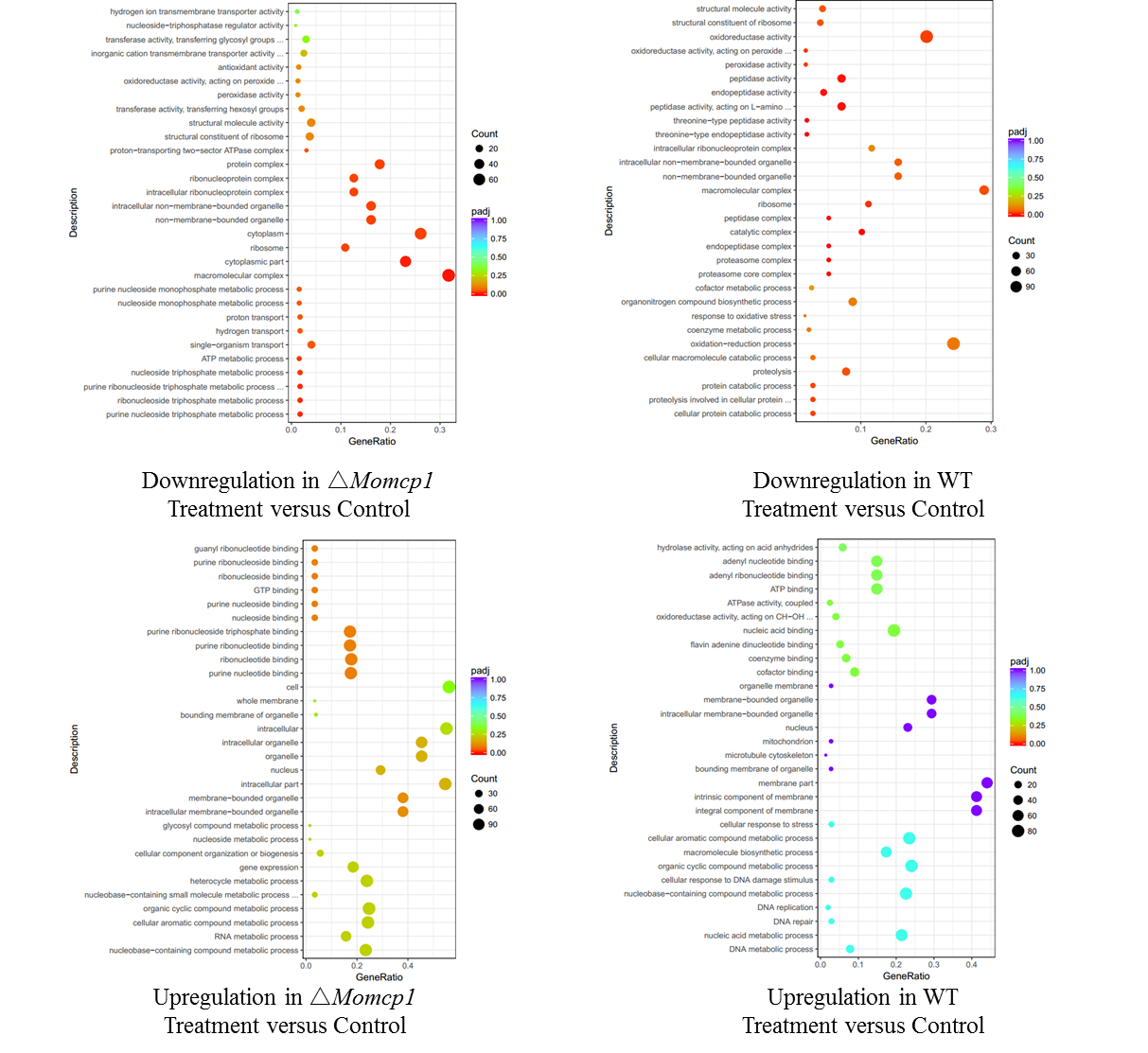


(**D**)

(**B**)

(**C**)

(**A**)

**Figure S6.** The GO enrichment analysis in WT and △*Momcp1* (**A**) Downregulation and (**B**) upregulation of pathways in △*Momcp1* under excessive Mn^2+^. (**C**) Downregulation and (**D**) upregulation of pathways in WT under excessive Mn^2+^.


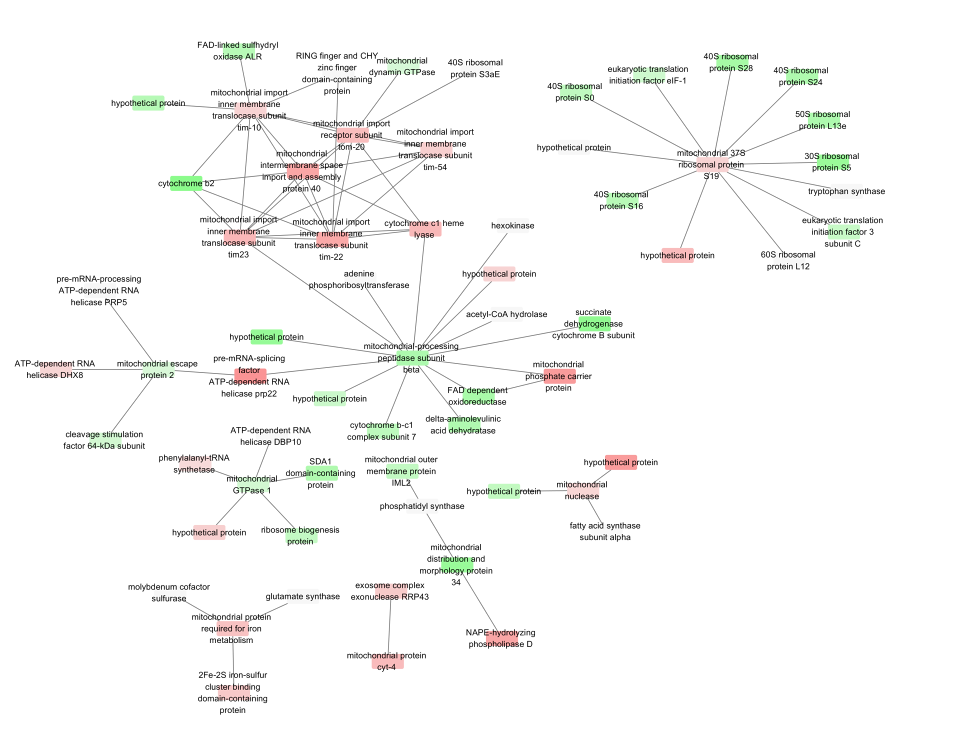

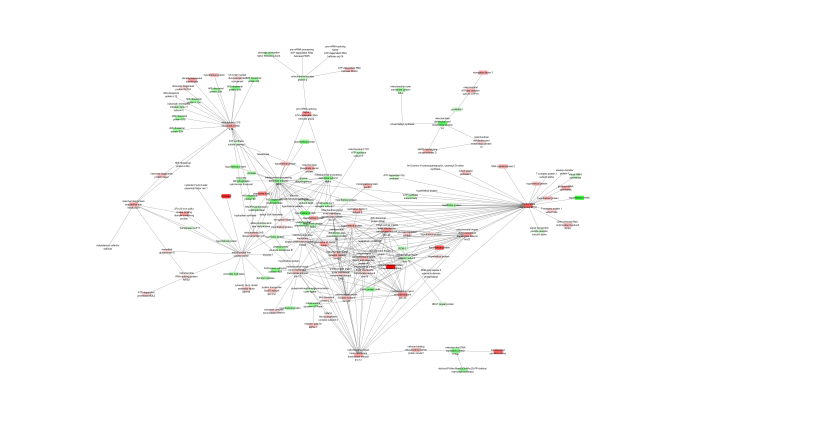


(**B**)

(**A**)

**Figure S7.** Protein and protein interaction network prediction for mitochondria related proteins in WT and △*Momcp1* mutant under excessive Mn^2+^. (A) WT, (B) △*Momcp1* mutant


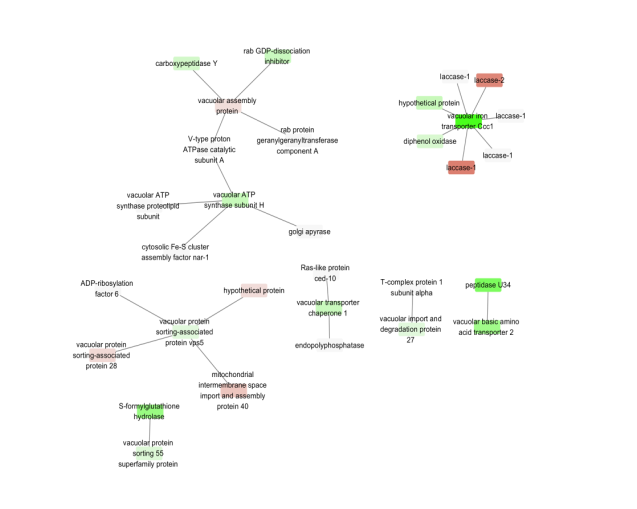

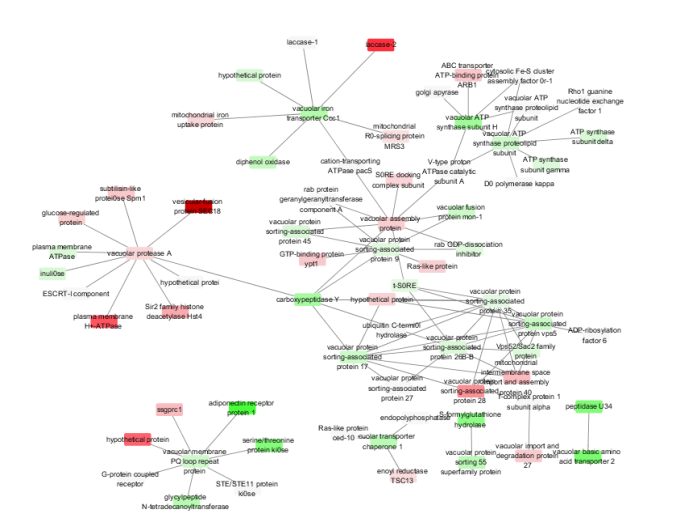


(**B**)

(**A**)

**Figure S8.** Protein and protein interaction network prediction for vacuole related proteins in WT and △*Momcp1* mutant under excessive Mn^2+^. (**A**) WT, (**B**) △*Momcp1* mutant
